# Randomized comparison of two commercial culture media (Cook and Vitrolife) for embryo culture after IMSI

**DOI:** 10.5935/1518-0557.20180058

**Published:** 2019

**Authors:** Claudia G. Petersen, Ana L. Mauri, Laura D. Vagnini, Adriana Renzi, Bruna Petersen, Mariana C. Matilla, Vanessa A. Comar, Juliana Ricci, Felipe Dieamant, João Batista A. Oliveira, Ricardo L.R. Baruffi, Jose G. Franco Jr

**Affiliations:** 1 Human Reproduction Prof. Franco Jr, Ribeirão Preto, Brazil; 2 Paulista Center for Diagnosis Research and Training, Ribeirao Preto, Brazil

**Keywords:** culture medium, MSOME, human embryo

## Abstract

**Objective:**

A variety of studies randomizing women/cycles or oocytes/embryos has been carried out to compare different culture media for culturing embryos up to cleavage or blastocyst stages showing controversial results. A recent systematic review suggested that data in the literature are insufficient to conclude the best culture medium for embryo quality, pregnancy and implantation. The objective of this study was to evaluate whether there is any difference between two commercial culture media regarding clinical outcomes after IMSI cycles.

**Methods:**

A total of 120 patients, ≤39 years of age, undergoing ART treatment submitted to the IMSI program were prospectively broken down and randomized into two groups: Group I (Cook media) and Group II (Vitrolife media).

**Results:**

Our data demonstrated that there was no difference using all the media from Cook or all the media from Vitrolife, for culturing embryos till day 2, in the bench incubator at low O2 concentration, in relation to fertilization, embryo quality, pregnancy and implantation rates (*p*>0.05).

**Conclusion:**

Both culture media used, Cook medium and Vitrolife medium, for the IMSI procedure and for later embryo culture with transfer on the second day, are equally effective and can be used depending on the ease and availability of acquisition.

## INTRODUCTION

Human assisted reproductive technology (ART) has been with us for over 4 decades now. The American and European registries of ART reported almost 700,000 cycles performed in the year of 2010. More than 5 million embryos have been cultured in different IVF labs around the world, and the consensus is that the culture medium (CM) plays a very important role in ART outcomes. Data collected over the past two decades by national IVF registers, suggests that *in vitro* cultured human embryos can be vulnerable to the CM composition (Market-Velker *et al*., 2010).

A great deal of scientific research and analysis have been applied to the development of a CM, which will successfully support in vitro growth, and development of human embryos through five to six days. Continuing research has led to numerous changes in the CM formulation, each with their advantages and disadvantages (Quinn, 2012). Actually, over 20 CM are commercially available and a variety of studies have shown satisfactory fertilization and embryo development using even a simple CM such as HTF or a very complex one (Biggers & Summers, 2008). In fact, the definition of which CM leads to the highest ART outcome is yet unknown in view that a trial comparing all CM simultaneously is impossible. A recent systematic review of only randomized CM (ran-CM) studies is insufficient to prove that one commercial CM is superior to the other due limited numbers and low methodological quality (Mantikou *et al*., 2013). The same group later on, in 2015 (Youssef *et al*., 2015), evaluating 32 studies, 17 studies randomized women (total 3666), three randomized cycles (total 1018) and 12 randomized oocytes (over 15,230) concluded that it was not possible to pool any of the data because each study compared different culture media. Most methodologies described in ran-CM papers, have not detailed the CM protocol they have applied, they describe the embryo culture, but nothing about the medium used during the all ART procedures, i.e. gamete and embryo culture media. The objective of our study was to compare two commercially available CM used for IMSI, in which all procedures; gamete (sperm and oocyte) preparation, IMSI, embryo culture and transfers were done by using supplemented media from Cook Medical compared to Vitrolife (Scandinavian IVF Science AB).

## MATERIAL AND METHODS

### Study Participants

A total of 120 patients, ≤39 years of age, undergoing ART treatment submitted to the IMSI program were divided prospectively and at random into two groups: Group I (Cook media) and Group II (Vitrolife media). Patient participation in each group was random, by drawing lots, using a randomization table previously elaborated for the study.

A total of 60 patients were included in Group 1 (Cook media) in which all procedures; gamete (sperm and oocyte) preparation, IMSI, embryo culture and transfers were done by using supplemented media from Cook Medical: 1) Gamete preparation: Sydney gamete buffer (for semen) and Sydney IVF fertilization (for oocyte); 2) IMSI procedures: Sydney gamete buffer and Sydney IVF PVP; 3) Embryo culture and transfer: Sydney IVF cleavage.

**Figure 1:**
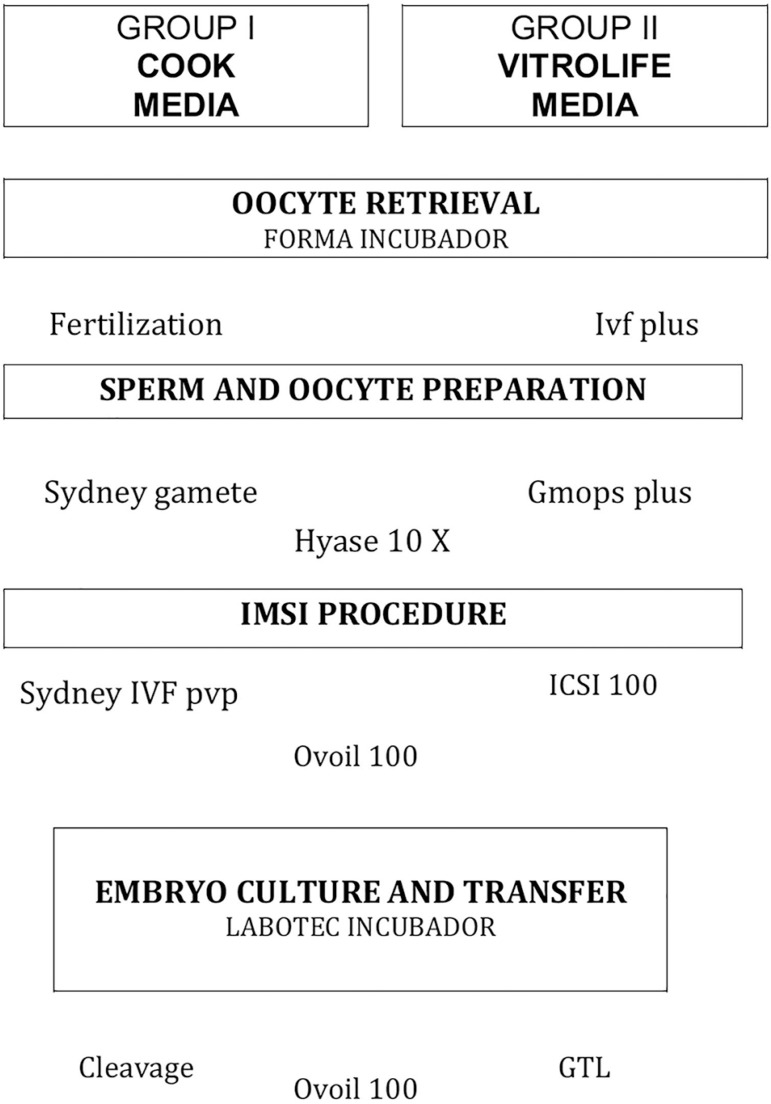
Media protocols used during all procedures

A total of 60 patients were included in Group 2 (Vitrolife media): All procedures; gamete (oocyte and sperm) preparation, IMSI, embryo culture and transfers were done by using supplemented media from Vitrolife (Scandinavian IVF Science AB): 1) Gamete preparation: GMOPS-plus (for semen) and GIVF-plus (for oocyte); 2) IMSI procedures: GMOPS-plus and PVP (ICSI-100); 3) Embryo culture and transfer: GTL media. All procedures (groups 1 and 2) were performed by using hyaluronidase (Hyase 10X) and oil (ovoil) from Vitrolife (Figure 1).

### Ovarian stimulation

All patients were submitted to the routine scheme of ovarian stimulation (Oliveira & Franco, 2016). Oocyte retrieval was performed 36h after human chorionic gonadotrophin (HCG) administration by transvaginal ultrasound-guided injection.

### Oocyte retrieval

The retrieved oocytes were incubated in CM (IVF fertilization/Cook) or (IVF plus/Vitrolife) at 37ºC and 5.5% CO_2_ for 1 hour. Cumulus cells were removed by exposing the oocytes to the Sydney gamete buffer/Cook or the GMOPS-plus/Vitrolife containing 80 IU/ml hyaluronidase (Vitrolife) for 30 sec., after which coronal cells were manually removed using denuding pipette by stripper (Cook, Australia). The denuded oocytes were classified according to their level of maturation. Oocytes with the first polar body, i.e., at the metaphase II (MII) stage, were considered mature and were used for the IMSI procedure

### Sperm preparation

Discontinuous gradients-Isolate (Cook) for Group I and Sperm-Prep-100TM (Scandinavian IVF Science AB, Sweden) for Group II-were used to separate the spermatozoa from the seminal fluid in the 40% and 90% fractions.

### IMSI procedure

A 1ml aliquot of sperm cell suspension was transferred to a 5 ml microdroplet of 7% polyvinyl-Instrument, USA Sydney gamete buffer/Cook or GMOPS-plus/Vitrolife, under sterile paraffin oil (Ovoil- 100, Vitrolife, Goteborg, Sweden). The sperm cells, suspended in the microdroplet, were placed on a microscope stage above an Uplan Apo 100x oil/1^35^ with the objective lens previously covered by a droplet of immersion oil. In this manner, suspended motile sperm cells in the observation droplet could be examined at high magnification through an inverted microscope (Eclipse TE 2000 U Nikon, Japan) equipped with high-powered differential interference contrast optics (DIC/Nomarski). The images were captured by a color video camera containing effective picture elements (pixels) for high quality image production and were projected onto a color video monitor. Morphological evaluation was accomplished on a monitor screen and the total calculated magnification was 15250 X (Total magnification: objective magnification 100x + camera video magnification + monitor magnification).

The spermatozoa used for IMSI were classified into 5 groups. Grade I consisted of spermatozoa free of any morphological abnormality (normal spermatozoa). A spermatozoon was classified as morphologically normal when it exhibited a normal nucleus as well as an acrosome, post-acrosomal lamina, neck, tail and mitochondria, besides not presenting a cytoplasmic droplet (Akl *et al*., 2011).

### Embryo culture

The injected oocytes were cultured in Cleavage^®^ media (Group I) or GTL^®^ media (Group II) individually, in microdrops of 50µl covered by oil, on Nunc dishes, in a bench incubator(Lab-CT/LabotecTM), with a pre-mixed gas (7%CO_2_/5%O_2_/88%N_2_), from day 0 to 2. The oocytes were examined after 17-20 h to assess fertilization and those with two distinct pronuclei were considered normal zygotes. Embryo quality was assessed at the time of transfer on day 2; the embryos were evaluated according to the following morphology criteria: Grade 0 (4 cells/symmetric/ without fragmentation), Grade 1 (no 4 cell and/or no symmetric cells with ≤25 fragmentation), Grade 2 (no symmetric cells with >25% fragmentation) and selected to be transferred.

## RESULTS

Our data demonstrated that there is no difference using the entire medium from Cook or the entire medium from Vitrolife, for culturing embryos until day-2 in the bench incubator at low O_2_ concentration, in relation of fertilization, embryo quality, pregnancy and implantation rates.

Patient age did not differ (*p*=0.1) between Group I (34.1±3.1) and Group II (35.1±3.6). The number of oocytes retrieved from Group I (9.5±5.8) was also similar (*p*=0.1) to that retrieved from Group II (8.6±5.6). In addition, there was no difference (*p*=0.9) in the number of oocytes retrieved at metaphase II between Group I (6.7±4.0) and Group II (6.1±4.5). Normal fertilization and cleavage rates were similar (*p*=0.7, *p*=0.6, respectively) for Group I (65.4±26.5 and 98.1±6.4, respectively) and Group II (65.8±20.5 and 97.2±10.2, respectively). Embryo quality (Grade 0, 1 and 2) was also similar (*p*=0.8, *p*=0.6, *p*=0.9, respectively) for both groups (Group I: 46% grade 0, 26% grade 1 and 28% grade 2) and (Group II: 40% grade 0, 38% grade 1 and 22% grade 2). There was no difference in the number of embryos transferred (*p*=0.07) between Groups I (1.8±0.5) and II (1.9±0.4). In addition, pregnancy rates/puncture, pregnancy rates/transfer and implantation rates, although higher for the cook media, did not show statistical difference between the two groups; Group I (46.7%, 51.9% and 33.3%, respectively) and Group II (38.3%, 41.1% and 26.4%, respectively). Table 1 summarizes the results.

**Table 1:** Results.

	GROUP I COOK MEDIA	GROUP II VITROLIFE MEDIA	
Nº of cycles	60	60	
Age (years)	34.1±3.1	35.1±3.6	0.1
Oocyte collected (n)	9.5±5.8	8.6±5.8	0.9
Metaphase II oocytes (n)	6.7±4.0	6.1±4.5	0.4
Normal fertilization rate(%)	65.4±26.5	65.8±0.5	0.7
Cleavage rate (%)	98.1±6.4	97.2±10.2	0.6
Total embryos	259	223	
Embryo quality (%) Grade 0 Grade 1 Grade 2	46%(119/259) 26%(68/259) 28%(72/259)	40%(89/223) 38%(85/223) 22%(49/223)	0.19 0.006 0.17
Embryo transferred (total) (n) Mean	102 1.8±0.5	106 1.9±0.4	0.07
Embryo quality transferred (%) Grade 0 Grade 1 Grade 2	69.6% (71/102) 24.5% (25/102) 5.9% (6/102)	66%(70/106) 25%(26/106) 9% (10/106)	0.68
Pregnancy rate (%) /puncture /transfer	46.7% (28/60) 51.9%(28/54)	38.3% (23/60) 41.1% (23/56)	0.46 0.33
Implantation rate (%)	33.3% (34/102)	26.4%(28/106)	0.29

## DISCUSSION

A number of commercial CM products are now available for procedures ranging from egg collection, semen preparation, embryo culture, embryo transfer and embryo cryopreservation. Commercial CM is "ready to use" with protein supplements added, and with other components and factors.

Nowadays, different formulations are available on market to be used from gamete preparation up to embryo development and implantation. The trouble is with the increasing of such many different formulations, presented commercially, the higher is the difficulty of knowing what the ideal formulation for embryo culture at the IVF lab was. Bartmann *et al*. (2016), applying a questionnaire in 37 Brazilian clinics to draw a profile of the most commonly used media, demonstrated that 25.93% of these clinics work with Vitrolife and 7.4% used the Cook CM. However, a great number of Brazilian clinics were not evaluated, and additional data is needed to confirm this information. The present study chose to compare Cook and Vitrolife CM, since they were commonly used in a lot in laboratories, including our laboratory, and for their ease of acquisition.

In this study, we performed a prospective and randomized analysis of 120 patients divided into two groups. From oocytes retrieval to embryo transfer and we used only Cook products for Group I and only Vitrolife products for Group II, so that the evaluation of the culture system would be as real as possible. Our results showed that the embryos cultured in Cook or Vitrolife CM systems were similar in their development and implantation. Normal fertilization (GROUP I COOK MEDIA: 65.4±26.5%; GROUP II VITROLIFE MEDIA: 65.8±20.5%), embryo cleavage (GROUP I COOK MEDIA: 98.1±6.4%; GROUP II VITROLIFE MEDIA: 97.2±10.2%) and embryo quality (GROUP I COOK MEDIA: Grade 0=46%, Grade 1=26%, Grade 2=28%; GROUP II VITROLIFE MEDIA: Grade 0=40%%, Grade 1=38%, Grade 2=22%) rates were almost the same (p>0.05). Although pregnancy rates/cycle (GROUP I COOK MEDIA: 46.7%; GROUP II VITROLIFE MEDIA:38.3%), per transfer (GROUP I COOK MEDIA: 51.9%; GROUP II VITROLIFE MEDIA:41.1%) and implantation rates (GROUP I COOK MEDIA: 33.3%; GROUP II VITROLIFE MEDIA:26.4%) were higher using Cook CM, they are not statistically significant (p>0.05).

In the literature, there are several studies comparing different commercial CM for IVF/ICSI with contradictory results, although most of them, basically describe the embryo CM but not the media used during all the procedure for embryo culture; i.e. from gamete preparation up to embryo development and has no IMSI procedure. Hoogendijk *et al*. (2007), comparing Sidney cook with Quinn's CM have showed better rates of embryo quality and pregnancy using Quinn's CM. A comparison between Sydney IVF and GM501 demonstrated significantly higher pregnancy rates with single a medium (Paternot *et al*., 2010). In addition, the comparison between the Maria Research center and Cook Sydney showed the same effectiveness when embryo quality and pregnancy rates were evaluated (Yoon *et al*., 2011). On the other hand, a comparison between IVF 50 (Vitrolife) and BM1 (Ellios) CM has described better embryo quality for those embryos cultured in BM1 CM (Parinaud *et al.*, 1998). However, when IVF CM was compared with P1 (Irvine) CM, Mauri *et al*. (2001) did not show differences between the media. Conversely, G5 (Vitrolife) had better blastocyst and pregnancy rates compared to Embryo Assisted (Medicult) CM.

Interesting, when two vitrolife (G1 and GTL) CM were compared to each other, similar results were found. In the present study, we evaluated the GTL CM from Vitrolife as the basis for this comparison. In fact, a specific comparison between Cook and Vitrolife CM has been poorly studied, especially GTL CM. Recently, Dumoulin *et al*. (2010) comparing pregnancy rates and perinatal outcomes from 188 single pregnancies found that clinical pregnancy rates, implantation rates, and mean birthweights were significantly lower in the cook group (K-SICM)compared with vitro life media group(G1 version 3). On the contrary, Carrasco *et al*. (2013) studied 449 patients and did not show any relationship between these two media media used for *in vitro* culture in terms of mean birthweight adjusted for gestational age and gender of singletons born after IVF/ICSI. However, to the moment, no comparison between GTL and Cook CM has been described. CM composition varies considerably, showing differences in pyruvate, lactate and amino acids, notably. Other factors such as physiological or environmental can also influence the results. Morbeck *et al*. (2014), evaluating animal embryos, demonstrated that CM composition together with protein supplements and oxygen concentration have a great influence in mouse blastocyst development. However, it is unknown in the literature whether human embryos are also affected by these interactions. In this study, we used in both groups i.e.; cook and vitrolife, supplementation and low oxygen to compare the effect of CM in outcomes. The presence of small studies with irrelevant controls, confounders and sub-optimal designs as well as the lack of detailed methodology, make it difficult to get any conclusion about which medium we should use. To better control, we should know the composition and preferably the formulation of each media we are using clinically. New formulations should have a scientific backing, standard minimum QC certificate by the companies, and more relevant tests such mega assay, genetic mouse assay which can monitor both early and late stage of embryo development.

## CONCLUSION

Both culture media used, Cook medium and Vitrolife medium, for the IMSI procedure and for later embryo culture with transfer on the second day were equally effective, and can be used depending on the ease and availability of acquisition.
